# Explicit Filtering Based Low-Dose Differential Phase Reconstruction Algorithm with the Grating Interferometry

**DOI:** 10.1155/2015/623236

**Published:** 2015-05-18

**Authors:** Xiaolei Jiang, Li Zhang, Ran Zhang, Hongxia Yin, Zhenchang Wang

**Affiliations:** ^1^Key Laboratory of Particle & Radiation Imaging (Tsinghua University), Ministry of Education, Beijing 100084, China; ^2^Department of Engineering Physics, Tsinghua University, Beijing 100084, China; ^3^Department of Radiology, Beijing Tongren Hospital, Capital Medical University, Beijing 100730, China; ^4^Department of Radiology, Beijing Friendship Hospital, Capital Medical University, Beijing 100050, China

## Abstract

X-ray grating interferometry offers a novel framework for the study of weakly absorbing samples. Three kinds of information, that is, the attenuation, differential phase contrast (DPC), and dark-field images, can be obtained after a single scanning, providing additional and complementary information to the conventional attenuation image. Phase shifts of X-rays are measured by the DPC method; hence, DPC-CT reconstructs refraction indexes rather than attenuation coefficients. In this work, we propose an explicit filtering based low-dose differential phase reconstruction algorithm, which enables reconstruction from reduced scanning without artifacts. The algorithm adopts a differential algebraic reconstruction technique (DART) with the explicit filtering based sparse regularization rather than the commonly used total variation (TV) method. Both the numerical simulation and the biological sample experiment demonstrate the feasibility of the proposed algorithm.

## 1. Introduction

X-ray grating interferometry [1–4] with conventional X-ray tubes develops rapidly in recent years and is becoming the most promising technology among various phase contrast imaging methods for clinical applications. Three kinds of information, that is, the attenuation, differential phase contrast (DPC), and dark-field images, can be obtained through one single scanning, and the latter two images provide additional and complementary information to the conventional attenuation image. The DPC method measures phase shifts of X-rays by obtaining the line integral of the directional derivatives of refractive index decrements (*δ*) [5], that is, the refraction angle of the beam. The refraction index reconstructed afterwards is 1000 times larger than the absorption index.

The phase-stepping approach of the grating interferometry, which requires a number of images to retrieve information, significantly increases the examine time and the dose delivered to the patient [6]. The problem becomes even more severe for DPC-CT because of the requirement of multiangle scanning. Therefore, reducing the number of projections, the exposure time, and the delivered dose is of great value. And that is why the low dose DPC reconstruction algorithm is proposed.

As mentioned above, the reconstruction problem for DPC-CT is to obtain the refraction index from the refraction angle data. The analytical method, the filtered backprojection (FBP) algorithm with the Hilbert transform, was first applied [7, 8]. Afterwards, several iterative algorithms, such as the maximum likelihood (ML) algorithm [9] and the differential algebraic reconstruction technique (DART) [10], were proposed. However, these algorithms rely on the completeness of data and the large number of projections.

The recently proposed compressed sensing (CS) theory [11] makes image reconstruction from incomplete data possible. Essentially, it illustrates that if the image is sparse in a domain which has small coherence with the sampling domain, according to the Shannon/Nyquist sampling theorem, fewer projections can almost accurately recover the images. A typical image reconstruction method exploits TV as the sparse regularization [12] (from CS measurements). Applications in both the absorption imaging [12] and the DPC imaging [10] have been implemented. Instead of the implicit regularization coming from the penalty, another sparse regularization method based on explicit filtering is proposed, which exploits spatially adaptive filters sensitive to image features and details [13]. However, no similar algorithms for DPC imaging have been suggested so far, which is just the problem to be solved in this paper.

In this work, we propose an explicit filtering based low-dose differential phase reconstruction algorithm. The algorithm combines the DART iterative algorithm and the explicit filtering based CS method. It has the potential to exactly reconstruct the refractive index distribution using few-view projections, thus reducing the exposure time and the delivered dose, making DPC-CT closer to clinical applications. The feasibility of the low dose reconstruction algorithm is verified by both the numerical simulation and the biological sample experiments.

## 2. Methods

### 2.1. Grating-Based Imaging

**Figure 1:**
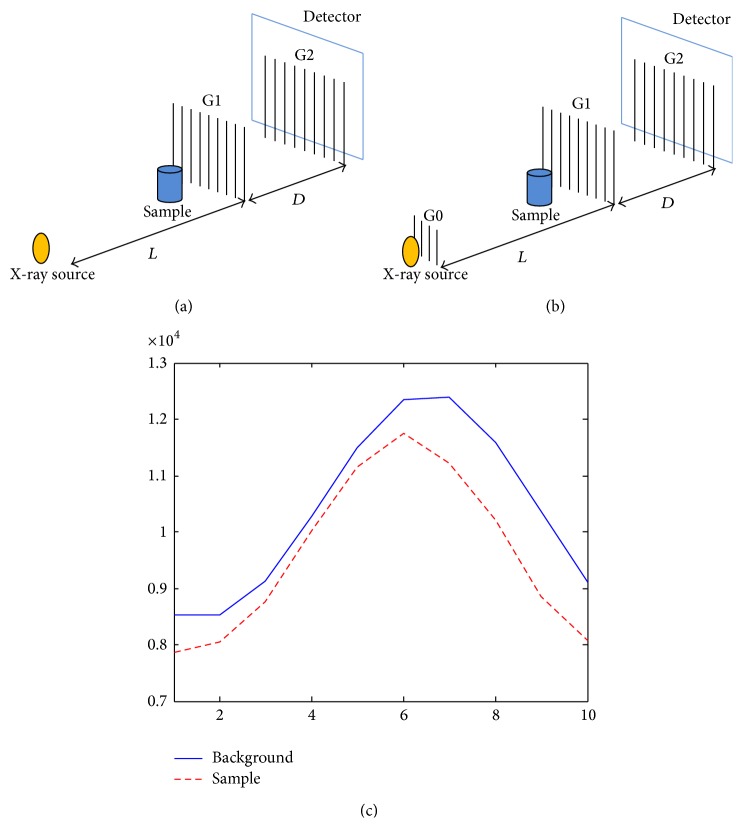
The grating interferometry. (a) Talbot effect based interferometry with coherent source; (b) Talbot-Lau effect based interferometry with incoherent source; the sample intensity oscillation curve (the dash line curve) and the background intensity oscillation curve (the solid line curve) measured with 10 steps during the phase stepping approach with the grating interferometer in Tsinghua University, China.

Figure 1 illustrates the schematic diagram of a typical grating interferometry. Two kinds of apparatuses are shown, the Talbot effect based interferometry with coherent source, that is, Figure 1(a), and the Talbot-Lau effect based interferometry with incoherent source, that is, Figure 1(b). The first grating G1 creates its self-image through Talbot-Lau effect or classical optics in the position of G2 where Moire fringes occur. The source grating G0 splits the source into an array of line sources, enabling the use of the large-focal-spot X-ray tube, that is, the incoherent source. The phase-stepping approach is adopted for image acquisition, capturing a series of raw images at every step of one of the gratings along the transverse direction, obtaining the intensity oscillation curve, Figure 1(c). The changes of the background oscillation curves determine three kinds of information, namely, the attenuation image, the DPC image, and the dark-field image.

To analyze the changes quantitatively, the oscillation curve for each pixel is expressed by the Fourier expansion series:

(1) Im,n,xg=∑iaim,ncos⁡2πxgp2+ϕim,n≈a0m,n+a1m,ncos⁡2πxgp2+ϕ1m,n,

where *a*_*i*_ is the amplitude coefficient, *ϕ*_*i*_ is the corresponding phase coefficient, and *p*_2_ is the period of G2. Then, the attenuation, dark-field, and DPC images are given by *T* = −log⁡⁡(*a*_0_^*s*^/*a*_0_^*r*^), *S* = −log⁡⁡(*V*_*s*_/*V*_*r*_), and DP = *ϕ*_1_^*s*^ − *ϕ*_1_^*r*^, respectively, where the superscripts (*s*) and (*r*) denote the values with the sample in place and as a reference without, respectively, and *V* = *a*_1_/*a*_0_ is the visibility of the oscillation curve.

### 2.2. Differential Phase-Contrast Reconstruction Algorithm

The DPC image measured by the grating interferometer is the refraction angle, which is related to the phase shift Φ(*r*) of the sample:

(2) $$\begin{array}{c} \text{DP}=\Delta \theta \approx \frac{\lambda }{2\pi }\frac{\partial \Phi \left(r\right)}{\partial r}. \end{array}$$

In other words, the grating interferometer obtains the line integral of the directional derivatives of refraction index decrements. Therefore, the reconstruction problem of DPC-CT can be expressed by

(3) $$\begin{array}{c} y_{\theta }=\int \frac{\partial \delta \left(i,j\right)}{\partial l^{\prime }}dl, \end{array}$$

where *y*_*θ*_ is the refraction angle projection, *δ* is the refraction index decrement of the samples, *l* is the path of X-ray beam in the medium and *l*′ is the perpendicular direction to *l*.

By contrast, the projection of the conventional X-ray transmission comes from the linear integration of attenuation coefficient; hence, the reconstruction problem can be expressed by

(4) $$\begin{array}{c} y_{I}=\int \mu \left(x,y\right)dl, \end{array}$$

where *y*_*I*_ is the intensity projection and *μ* is the linear attenuation coefficient.

Such difference in mathematical expressions of the projections requires different reconstruction algorithms.

Inspired by the widely used algebraic reconstruction technique (ART) [14], Wang et al. proposed a differential algebraic reconstruction technique (DART) by discretizing the projection process of the differential phase contrast imaging into a linear partial derivative matrix [10]:

(5) $$\begin{array}{c} y_{\theta }=\int \frac{\partial \delta \left(i,j\right)}{\partial l^{\prime }}dl\approx \sum \frac{\partial \delta \left(i,j\right)}{\partial l^{\prime }}=\langle b_{i},x\rangle , \end{array}$$

where *b*_*i*_ denotes the net interpolation coefficient corresponding to each pixel.

Therefore, the forward projection process of differential phase contrast imaging can be expressed by

(6) $$\begin{array}{c} \Delta \theta =Bx, \end{array}$$

where *B* is named as the linearly partial-derivative matrix. Equation 3 can be used to reconstruct the refractive index by algorithms similar to ART directly shown as 4:

(7) $$\begin{array}{c} x^{k+1}=x^{k}+\frac{y_{\theta }-Bx^{k}}{\left\|B\right\|}B^{T}, \end{array}$$

where *x* ^*k*^ is the image vector *x* in the *k*th iteration; *Bx* ^*k*^ presents the forward projection process.

However, the DART algorithm relies on the completeness of data [10]. It is incompetent at the ill-posed reconstruction problems, such as the cases of few-view or limited-angle projections.

### 2.3. Explicit Filtering Based Low-Dose Differential Phase-Contrast Reconstruction Algorithm

In this part, we propose an explicit filtering based low-dose differential phase-contrast reconstruction algorithm based on the above DART algorithm for DPC reconstruction.

Generally, the compressed sensing DPC-CT reconstruction method can be summarized as

(8) $$\begin{array}{c} \hat{x}=\underset{x}{\arg\;\min}\left\{{\left\|y_{\theta }-Bx\right\|}^{2}+\lambda \varphi \left(x\right)\right\}.\; \end{array}$$

Here, the fitness function ‖*y*_*θ*_ − *Bx*‖^2^  , which tries to match the estimation to the data, is accomplished by the DART algorithm above, where the operation ‖·‖ represents the *l*-2 norm. The regularization function *φ*(*x*) expresses some priori known property of the unknown object, which usually defines a sparse representation of *x* after a specific transformation. The regularization parameter *λ* balances the fitness function with the regularization function. Different regularization methods are used to find the solution of the mathematic model under different constraints of minimizing the complexity of reconstructed signals in different representations. The typical TV-type regularization method uses the total variations as the sparse transformation, which is a parametric regression technique. Another solution is to replace the parametric regression by spatially adaptive filters, which is sensitive to image features and details. In this paper, the BM3D filter is adopted, that is, DART_BM3D algorithm.

**Figure 2:**
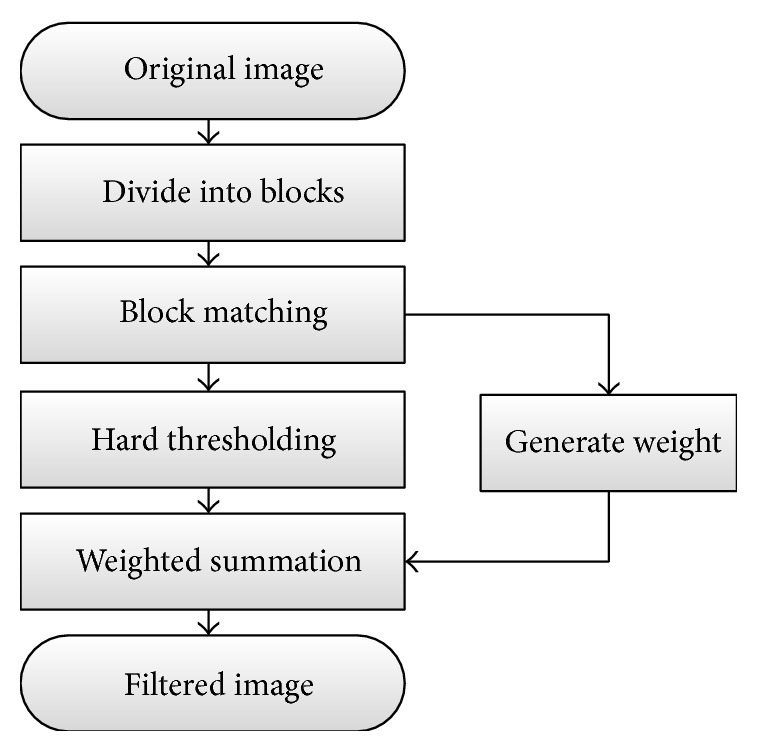
The flow chart of BM3D filter.

The BM3D algorithm is based on an enhanced sparse representation in transform domain [15]. The enhancement of the sparsity is achieved by grouping similar 2D fragments of the image into 3D data arrays. And then the collaborative filtering with the hard thresholding is carried out, which enhances the similarity between the blocks while at the same time preserves even the finest details with their essential unique features shared by the jointly filtered 2D fragments. The implementation of BM3D filter is shown as Figure 2.

BM3D filter matches the similar block regions in the image (block matching, BM) using the similarity defined as

(9) $$\begin{array}{c} d\left(Z_{1}-Z_{2}\right)=\frac{{\left\|Z_{1}-Z_{2}\right\|}_{2}^{2}}{N_{\text{mat}}^{2}}, \end{array}$$

where *Ζ* is the numerical metric of the image, *N*_mat_ is the side length of block, and *d*(*Z*_1_ − *Z*_2_) is the difference between two blocks.

Blocks with small differences can be grouped as a set *S*. For blocks in the same group, a hard threshold is used to assign zeros for small value pixels to generate a weight for blocks:

(10) $$\begin{array}{c} w_{Z}^{S}=\left\{\begin{array}{cc} \frac{1}{\sigma^{2}N_{\text{har}}^{2}} & N_{\text{har}}\neq 0\\ 1 & N_{\text{har}}=0, \end{array}\right. \end{array}$$

where *N*_har_ is the number of nonzero pixels. Then, the filtered image can be obtained as a similarity-weighted average:

(11) $$\begin{array}{c} \lambda \left(x\right)=\frac{\sum_{x\in Z}\sum_{Z\in S}w_{Z}^{S}Z\left(x\right)}{\sum_{x\in Z}\sum_{Z\in S}\chi \left(x\right)},\;\chi \left(x\right)=\left\{\begin{array}{cc} 1 & Z\left(x\right)\neq 0\\ 0 & Z\left(x\right)=0. \end{array}\right. \end{array}$$

**Figure 3:**
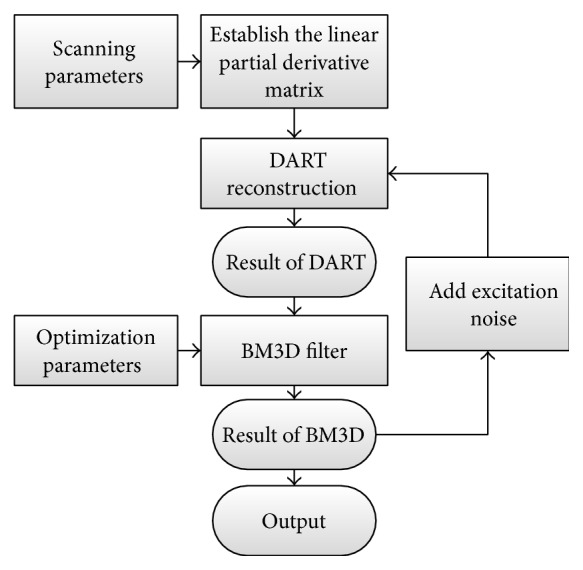
The flow chart of the explicit filtering based low-dose differential phase-contrast reconstruction algorithm.

The process of our reconstruction algorithm is shown in Figure 3 and the steps are described as follows.

(A) Initialization. Creating the linearly partial-derivative matrix *B* for few-view scanning based on the geometry parameters. An initial guess of the reconstruction image ${\hat{x}}_{0}=0$ is given and *n* = 1.
(B) DART reconstruction:
  (12) $$\begin{array}{c} {\hat{x}}^{k+1}=\text{DART}\left({\hat{x}}_{k}\right), \end{array}$$
  where DART represents the reconstruction method in 4.
(C) BM3D filter:
  (13) $$\begin{array}{c} {\hat{x}}_{k+1}=\text{BM}3\text{D}\left({\hat{x}}^{k+1}\right), \end{array}$$
  where BM3D represents the method shown as in Figure 2.
(D) Add excitation noise into ${\hat{x}}_{k+1}$ and go back to step (B).

Gaussian noise is added in the unobserved portions, that is, the missing angles caused by the few-view or limited-angle scanning, in frequency domain, which works as a random generator of the missing components in the spectrum:

(14) $$\begin{array}{c} {\hat{x}}_{k+1}=\text{FF}{\text{T}}^{-1}\left(\text{FFT}\left({\hat{x}}_{k+1}\right)+\text{Gaussian}\left(0,\sigma \right)\right), \end{array}$$

where FFT represents the Fourier transform operation and FFT^−1^ is the inverse Fourier transform operation, *σ* is the standard deviation of the Gaussian noise which is determined empirically by the noise of the projections caused by the quantum noise and electronics noise.

## 3. Experiments and Results

The proposed explicit filtering based low-dose differential phase-contrast reconstruction algorithm was validated by both the numerical simulation and biological experiments.

### 3.1. Numerical Simulation

**Figure 4:**
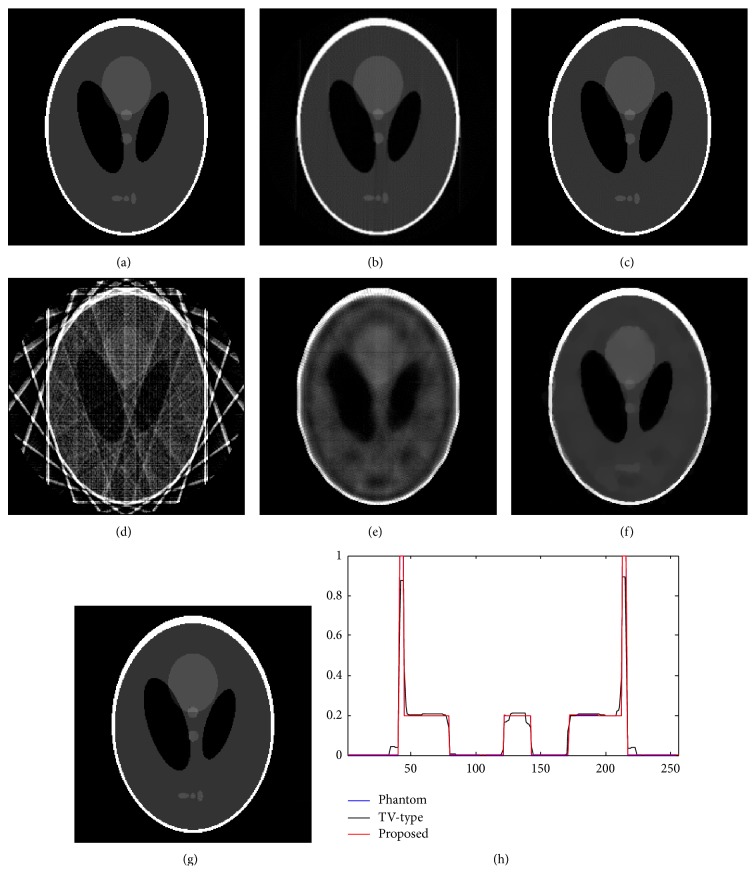
The reconstruction results. (a) The phantom; (b) the result of FBP with 180 views, MSE = 4.51*e* − 3; (c) the result of DART with 180 views, 500 iterations, MSE = 2.84*e* − 5; (d) the result of FBP with 10 views, MSE = 5.15*e* − 2; (e) the result of DART with 10 views, 2000 iterations, MSE = 5.80*e* − 3; (f) the result of DART_TV with 10 views, 2000 iterations, MSE = 1.63*e* − 3; (g) the result of the proposed algorithm with 10 views, 2000 iterations, MSE = 4.62*e* − 6; (h) the profiles of the 128th line of (a), (f), and (g).

The Shepp-Logan phantom with the resolution of 256 × 256 was used in the study. The phantom is shown as in Figure 4(a) and the reconstructed results are shown as in Figures 4(b)–4(g). All images are shown in the same display window [0,1].

Figures 4(b) and 4(c) are the reconstructed results with the FBP and DART (500 iterations) methods, respectively, with 180 views within 180° with 1° angular interval, which verified the effectiveness of the FBP and DART methods in case of complete data. Figures 4(d) and 4(e) are the reconstructed results with the FBP and DART, respectively, methods with 10 views within 180° with 18° angular interval, with an angular downsampling factor of 18. The results demonstrate the dependency on the completeness of the data of the FBP and DART (2000 iterations) methods.

Figure 4(f) is the reconstructed result with the typical TV-type compressed sensing method (2000 iterations). The method is an effective reconstruction method dealing with incomplete data.  Figure 4(g) is the reconstructed results with proposed explicit filtering based compressed sensing methods (2000 iterations), which is also skilled in dealing with incomplete data. The reconstructed results with the compressed sensing methods are almost artifact-free and they are in high accordance with the phantom in both appearance and values, while the results of FBP and DART algorithms show severe streaking artifacts due to the downsampling. Furthermore, profiles as shown in Figure 4(h) show that the proposed method is better than the TV-type method in preserving details.

### 3.2. Biological Sample Experiment

The biological sample results have been used to test the proposed reconstruction method. The experiments were performed at the TOMCAT beamline using a two-grating interferometer operated at 25 KeV and in the 3rd Talbot distance at the Swiss Light Source of the Paul Scherrer Institute in Switzerland. The pitch of the phase grating *p*_1_ was 3.981 *μ*m with a height of *h*_1_ = 31.7 *μ*m. The corresponding values for the second grating (gold absorber grating) were *p*_2_ = 2.00 *μ*m and *h*_2_ = 24 *μ*m. The sample was a guinea pig eyeball packed in a plastic pipe and was scanned within 180° with equivalent angular interval of 1°. For each view, an eight-step phase stepping process was adopted and the refraction angular projections were retrieved by information retrieving algorithm.

**Figure 5:**
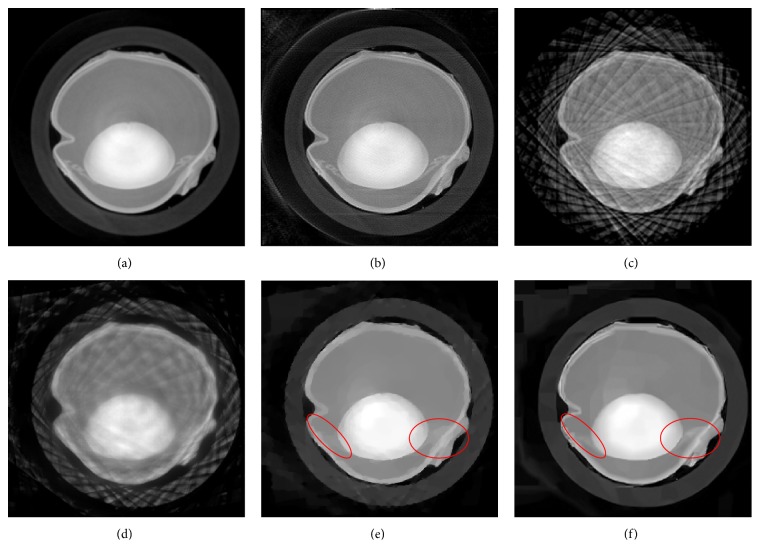
The reconstruction results. (a) the result of FBP with 180 views; (b) the result of DART with 180 views; (c) the result of FBP with 10 views; (d) the result of DART with 20 views; (e) the result of DART_TV with 20 views; (f) the result of the proposed algorithm with 20 views.

**Figure 6:**
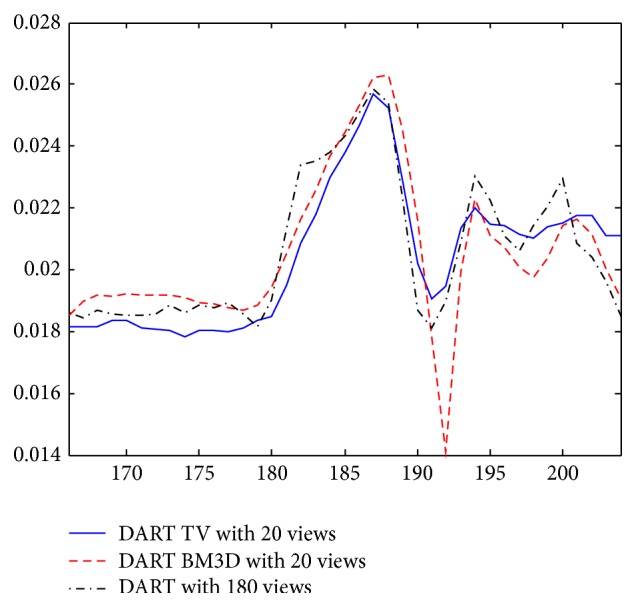
The profiles of the 188th line in the red circles on the right.

The reconstruction results are shown in Figure 5. Figures 5(a) and 5(b) are the results of FBP algorithm and DART algorithm with 180 views, respectively. Since the reconstruction image is more complex than the phantom in the previous section, an angular down-sampling factor of 9 was adopted. Figures 5(c) and 5(d) is the results of FBP algorithm and DART algorithm with 20 views, respectively. The results show severe streaking artifacts, especially the FBP result. As shown in Figures 5(e) and 5(f), the results of the two compressed sensing methods are nearly artifact-free. Both the results of the two compressed sensing methods show great image quality improvement compared with DART and FBP in few-view reconstruction. And the red ellipses in Figures 5(e) and 5(f) illustrate the better capabilities of preserving details of the proposed explicit filtering based method than the TV-type method, which can also be seen from Figure 6, which shows the profiles of the 188th line in the red circles on the right.

## 4. Conclusions

In this paper, an explicit filtering based low-dose differential phase-contrast reconstruction algorithm is proposed. The algorithm is an application of the compressed sensing theory and has the potential to accurately reconstruct the distribution of the refractive index with few-view projections.
